# A Dilemma in the Diagnosis of Group A Streptococcus (GAS) Meningitis Versus Drug-Induced Aseptic Meningitis: A Case Report

**DOI:** 10.7759/cureus.79443

**Published:** 2025-02-22

**Authors:** Rahul Sharma, Nadim Jaafar, Navami Guru, Edward Oh, Siddartha Guru

**Affiliations:** 1 Internal Medicine, Greater Baltimore Medical Center, Towson, USA; 2 Radiology, Greater Baltimore Medical Center, Towson, USA; 3 Infectious Diseases, Penn State Health Milton S. Hershey Medical Center, Hershey, USA

**Keywords:** a case report, adult bacterial meningitis, drug-induced aseptic meningitis, group a streptococcus pyogenes, invasive group a streptococcus infections

## Abstract

Group A *Streptococcus* (GAS), also called *Streptococcus pyogenes*, is a rare cause of adult meningitis. In recent years, many outbreaks of invasive *S. pyogenes* infections in Europe, involving the *emm*1.0 subtype of the M1UK lineage, have led to a rising prevalence of GAS meningitis. We present a case with a diagnostic dilemma involving a 55-year-old female with otitis media, *S. pyogenes* bacteremia, and symptoms of meningitis. However, cerebrospinal fluid (CSF) tests were not completely consistent with bacterial meningitis, with normal glucose levels on CSF analysis, no organism seen on gram stain, and no growth on CSF culture. Drug-induced aseptic meningitis (DIAM) was considered, given the patient's use of ibuprofen prior to admission, and thus, non-steroidal anti-inflammatory drugs (NSAIDs) were avoided during her hospitalization. The GAS bacteremia was treated with intravenous ampicillin. She improved a few days later, but we are uncertain whether the antibiotics or the avoidance of NSAIDs resolved her meningitis symptoms. On discharge, ampicillin was switched to intravenous ceftriaxone, 2 g every 12 hours, to treat possible GAS meningitis for a four-week duration.

## Introduction

Group A *Streptococcus* (GAS), also known as *Streptococcus pyogenes*, is a gram-positive, beta-hemolytic bacterium that is present on the skin and mucosal surfaces. It commonly causes pharyngitis, skin and soft tissue infections, and otitis media, but can cause invasive infections such as necrotizing fasciitis, bacteremia, and, rarely, meningitis [1]. Invasive GAS infections, including meningitis, have risen over the past two decades in the US, with approximately 9,700 cases in 1997 to about 27,400 cases in 2022 [2]. There is no surveillance data specifically for GAS meningitis currently in the US, but the prevalence of GAS meningitis has been increasing worldwide [3,4]. Drug-induced aseptic meningitis (DIAM) can present similarly to bacterial meningitis, with symptoms of fever, meningeal symptoms, headaches, and altered mental status. Cerebrospinal fluid (CSF) studies usually show numerous neutrophils and elevated protein, making it difficult to differentiate from bacterial meningitis.

## Case presentation

A 55-year-old female with a history of hypertension and paroxysmal atrial fibrillation developed right-sided ear pain and fullness about two weeks prior to admission. About 10 days prior to admission, she noticed mild hearing difficulties and, hence, presented to urgent care, where she was diagnosed with otitis media and cerumen impaction. She was prescribed oral amoxicillin and ofloxacin ear drops. Despite being on antibiotics for one week, she had worsening symptoms, including a new onset headache. Her primary care physician referred her to ENT and asked her to continue the antibiotics. The patient reported consuming excessive amounts of ibuprofen to control her headaches and ear pain. The day prior to admission, she became confused with progressively worsening lethargy; her husband was concerned, so he brought her to the Emergency Department (ED) for further evaluation.

In the ED, she was afebrile, normotensive, and tachycardiac (Table 1), and on physical examination, she was alert and oriented but slow to respond to questions. She had decreased hearing in the right ear, and on evaluation with an otoscope, there was debris and drainage in the right external auditory canal. A limited view of the tympanic membrane was noted, but she was able to discern mild erythema of the tympanic membrane. A few hours later, in the ED, she developed a fever of 39.2°C, triggering a full sepsis work-up. At the time, she denied photophobia or neck stiffness. Labs revealed leukocytosis with bandemia, mild anemia, and normal lactic acid and creatinine levels (Table 2).

**Table 1:** Vitals on admission

Vitals	Values
Temperature	37.7ºC
Blood pressure	144/86
Heart rate	109 heartbeats per minute
Respiratory rate	16 breaths per minute
SpO2	97% on room air

**Table 2:** Labs on admission

Labs	Values	Reference
White blood cell count	33.64 x 10^3^/uL	4.00 - 11.00 x 10^3^/uL
Bands manual	37.8%	3 - 17%
Hemoglobin	12.4 g/dL	12.5 - 15.0 g/dL
Platelets	290 x 10^3^/uL	150 - 450 x 10^3^/uL
Creatinine	0.56 mg/dL	0.40 - 1.10 mg/dL
Lactic acid	1.4 mmol/L	0.5 - 1.9 mmol/L

Two sets of peripheral blood cultures were drawn prior to broad-spectrum antibiotics for empiric meningitis coverage with ceftriaxone, vancomycin, acyclovir, and ampicillin, together with dexamethasone. Less than 30 minutes after initiating antibiotics, a lumbar puncture was performed. The CSF showed 7188/cumm white blood cells (WBCs) with 96% neutrophilic predominance, elevated glucose of 96 mg/dL, and elevated protein of 164 mg/dL (Table 3). The serum glucose level at the time of lumbar puncture was 128 mg/dL. The CSF gram stain was negative. In addition to normal glucose levels, these findings were consistent with aseptic meningitis, particularly DIAM, given the use of ibuprofen prior to admission. Thus, a recommendation was made to hold any further non-steroidal anti-inflammatory drugs (NSAIDs) and continue empiric antibiotics until further CSF studies resulted.

**Table 3:** CSF results including cultures CSF, Cerebrospinal Fluid; WBCs, White Blood Cells; RBCs, Red Blood Cells; HSV PCR, Herpes Simplex Virus Polymerase Chain Reaction; VZV PCR, Varicella Zoster Virus Polymerase Chain Reaction; IgM, Immunoglobulin M; IgG, Immunoglobulin G; VDRL, Venereal Disease Research Laboratory; PMNs, Polymorphonuclear Leukocytes

CSF studies	Result	Normal values	Interpretation
Glucose	96 mg/dL	40 - 70 mg/dL	High
Total protein	164.4 mg/dL	15 - 45 mg/dL	High
WBC	7188/cumm	0 - 6/cumm	High
RBC	0/cumm	0/cumm	Normal
Neutrophil %	96%	0%	High
HSV PCR	Not detected	-	-
VZV PCR	Not detected	-	-
West Nile antibodies IgM	IgM < 0.6	IgM < 0.6	Normal
West Nile antibodies IgG	IgG < 1.3	IgG < 1.3	Normal
VDRL	Non-reactive	-	-
Gram-stain	No organisms or PMNs	-	-
Bacterial culture	No growth	-	-
Fungal culture	No growth	-	-
Acid-fast bacilli culture	No growth	-	-

**Figure 1:**
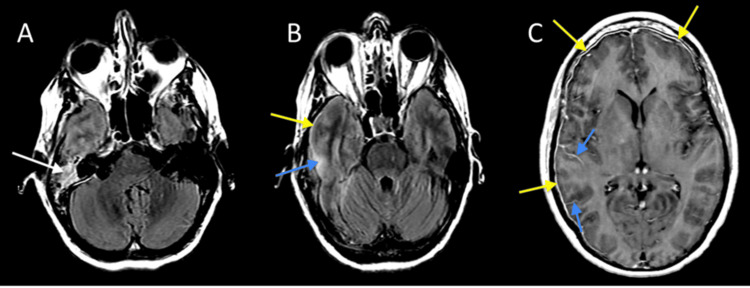
MRI brain with contrast (A) Axial T2 FLAIR MRI of the brain at the level of the temporal bones demonstrates right mastoid-middle ear hyperintensity (white arrow), consistent with otomastoiditis. (B) Axial T2 FLAIR MRI of the brain just superior to Figure 1A shows pachymeningeal thickening with hyperintensity (yellow arrow), consistent with pachymeningitis, sulcal, with adjacent temporal lobe parenchymal hyperintensity (blue arrow), indicating leptomeningitis and early cerebritis. (C) Axial contrast-enhanced T1-weighted MRI (Figures 1A-1B) demonstrates pachymeningeal (yellow arrows) and leptomeningeal (blue arrows) enhancement over the cerebral hemispheres, indicating pachymeningitis and leptomeningitis. FLAIR MRI, Fluid-Attenuated Inversion Recovery Magnetic Resonance Imaging

Overnight, she remained febrile, and early in the morning on hospital day (HD) 2, she had worsening confusion and concern for right-sided weakness. The stroke response team was activated; the computed tomography (CT) of the head without contrast and CT perfusion of the head and neck showed no evidence of infarction, acute bleed, mass lesion, or large vessel occlusions. Magnetic resonance imaging (MRI) of the brain, with and without contrast, done later that day, showed complicated right mastoiditis/otitis with suspected adjacent early right temporal cerebritis and findings raising the possibility of asymmetric leptomeningitis and pachymeningitis/reactive dural thickening, right greater than left, with no empyema or abscess (Figures 1A-1C). She had no right-sided weakness a few hours later that day on the exam but continued to be confused. The Lyme serologies, Legionella, and streptococcal urine antigens were all negative.

On HD 3, the CSF cultures remained negative, but blood cultures from admission grew ampicillin-sensitive *S. pyogenes*, with susceptibilities as seen in Table 4. The antibiotics were narrowed to intravenous ampicillin 2 g every four hours and acyclovir. Repeat blood cultures were obtained.

**Table 4:** Streptococcus pyogenes susceptibilities

Antibiotics	Minimum inhibitory concentration (MIC)	Result
Amoxicillin	<=0.25 mcg/mL	Susceptible
Cefepime	<=0.0625 mcg/mL	Susceptible
Cefotaxime	<=0.0625 mcg/mL	Susceptible
Ceftriaxone	<=0.0625 mcg/mL	Susceptible
Clindamycin	0.0625 mcg/mL	Susceptible
Erythromycin	0.0625 mcg/mL	Susceptible
Levofloxacin	1.0 mcg/mL	Susceptible
Linezolid	1.0 mcg/mL	Susceptible
Meropenem	<=0.0625 mcg/mL	Susceptible
Penicillin	<=0.03125 mcg/mL	Susceptible
Vancomycin	0.5 mcg/mL	Susceptible

On HD 4, CSF herpes simplex virus polymerase chain reaction (HSV PCR), varicella zoster virus polymerase chain reaction (VZV PCR), and enterovirus PCR were all negative, so the acyclovir was stopped. Her mental status improved and she was no longer febrile.

On HD 5, a transesophageal echocardiogram (TEE) to rule out endocarditis showed no evidence of vegetation, and repeat blood cultures from HD 3, at 48 hours, were negative.

In anticipation of discharge, the ampicillin was switched to intravenous ceftriaxone 2 g every 12 hours for the convenience of less frequent dosing, with a plan for a total duration of four weeks of antibiotics from negative blood cultures, due to concern for GAS meningitis/cerebritis based on clinical suspicion and imaging, even though the CSF studies were not suggestive of bacterial meningitis/cerebritis.

## Discussion

GAS meningitis has historically been a rare entity in the US [5]. However, in recent years, there has been an upsurge of invasive GAS infections, including meningitis. Denmark and the Netherlands have reported a surge in GAS infections from a specific M1UK lineage of the *emm*1.0 subtype since 2022 [3,4]. The origin of the M1UK lineage is believed to have arisen as a consequence of the 2008 pharyngitis guidelines in the UK [6]. Over half of the cases between January and March 2023 in the UK were from the *emm*1.0 strain, with 90% caused by the M1UK variant lineage. In the US, isolates from 122 invasive GAS-reported infections in 2022 were of the *emm*1.0 subtype [2]. The Centers for Disease Control and Prevention (CDC) data does not specify if these are of the M1UK variant; however, given the global trend, it is a possibility. Our patient could have had the *emm*1.0 subtype M1UK variant strain, but we were unable to confirm this, given the limited testing availability at our facility.

The dilemma in this case stems from the discordance between the clinical presentation and the microbiological and laboratory data. The evidence supporting meningitis from GAS included the following: (1) the clinical presentation of right-sided otitis media/externa from GAS, which is consistent with previous GAS meningitis infections, where 43% of the cases were preceded by acute otitis media [7]; (2) the MRI shows adjacent right temporal cerebritis, asymmetric leptomeningitis, and pachymeningitis/reactive dural thickening; (3) the presence of invasive GAS infection with bacteremia; and (4) some CSF studies supported bacterial meningitis, including the elevated WBC count with neutrophilic predominance and high protein count.

The evidence that supports the possibility of an alternative cause of meningitis, such as DIAM, included the following: (1) CSF glucose was elevated to 96, while serum glucose levels at the time of lumbar puncture were 128 mg/dL; (2) CSF gram stains showed no organisms, and there was no growth on CSF cultures despite the patient having GAS bacteremia at the same time and intravenous antibiotics being initiated only 30 minutes before the lumbar puncture; and (3) the patient reported consuming a large volume of high-dose ibuprofen prior to admission.

DIAM is a rare cause of meningitis, with fewer than 200 cases reported [8]. Patients usually present with fevers, headaches, meningeal signs, nausea, vomiting, and altered mental status [9]. CSF findings may vary, though they more commonly reveal polymorphonuclear pleocytosis (though lymphocytic and eosinophilic predominance can also be present), variably high protein levels but normal glucose levels, and negative CSF gram stain and cultures [10]. NSAIDs are the most common cause of DIAM, accounting for about 32% of the cases, with ibuprofen being the most common among the NSAIDs [10,11]. There are multiple hypotheses on the mechanisms by which DIAM occurs with different medications. NSAIDs are postulated to combine with a CSF protein, which acts as a hapten, triggering an inflammatory response limited to the meninges [12]. Treatment involves drug cessation, which helps resolve symptoms within 2-14 days [11].

A case report on ibuprofen-induced DIAM noted that rechallenging patients with ibuprofen resulted in generalized malaise, headache, chills, rigors, fevers, and nuchal rigidity within hours. Cessation of the NSAIDs, together with treatment with methylprednisolone and Tylenol, resulted in the resolution of symptoms within 24 hours [13].

In our case, empiric antibiotics were started to cover meningitis, but CSF studies were consistent with aseptic meningitis. Concerned about possible DIAM from ibuprofen ingested by the patient, NSAIDs were avoided throughout the hospitalization. Higher doses of antibiotics were also used, which would cover bacterial infections in the central nervous system. The patient's mental status improved after one week of antibiotics and discontinuation of NSAIDs. Since the interventions were introduced at the same time, the reason for the clinical improvement is difficult to pinpoint. Future use of ibuprofen and meningitis-like symptoms in this patient would confirm that this episode was likely DIAM.

## Conclusions

Historically, GAS meningitis has been rare, but there has been a recent surge globally, which is hypothesized to be secondary to the *emm*1.0 M1UK variant. DIAM can present similarly to bacterial meningitis, especially in patients who have received ibuprofen, which is the most common cause of DIAM. Our case highlights the importance of keeping a broad differential when evaluating patients for meningitis.
